# Genomic insights of protein arginine methyltransferase Hmt1 binding reveals novel regulatory functions

**DOI:** 10.1186/1471-2164-13-728

**Published:** 2012-12-26

**Authors:** Eric J Milliman, Zihua Hu, Michael C Yu

**Affiliations:** 1 Department of Biological Sciences, State University of New York at Buffalo, Buffalo, NY, 14260, USA; 2 Center for Computational Research, New York State Center of Excellence in Bioinformatics & Life Sciences, Department of Ophthalmology, Department of Biostatistics, Department of Medicine, State University of New York at Buffalo, Buffalo, NY, 14260, USA; 3 SUNY Eye Institute, Buffalo, NY, 14260, USA

**Keywords:** Protein arginine methylation, Hmt1, RNA Pol III transcription, tRNA biogenesis, ChIP-chip

## Abstract

**Background:**

Protein arginine methylation is a post-translational modification involved in important biological processes such as transcription and RNA processing. This modification is catalyzed by both type I and II protein arginine methyltransferases (PRMTs). One of the most conserved type I PRMTs is PRMT1, the homolog of which is Hmt1 in *Saccharomyces cerevisiae*. Hmt1 has been shown to play a role in various gene expression steps, such as promoting the dynamics of messenger ribonucleoprotein particle (mRNP) biogenesis, pre-mRNA splicing, and silencing of chromatin. To determine the full extent of Hmt1’s involvement during gene expression, we carried out a genome-wide location analysis for Hmt1.

**Results:**

A comprehensive genome-wide binding profile for Hmt1 was obtained by ChIP-chip using NimbleGen high-resolution tiling microarrays. Of the approximately 1000 Hmt1-binding sites found, the majority fall within or proximal to an ORF. Different occupancy patterns of Hmt1 across genes with different transcriptional rates were found. Interestingly, Hmt1 occupancy is found at a number of other genomic features such as tRNA and snoRNA genes, thereby implicating a regulatory role in the biogenesis of these non-coding RNAs. RNA hybridization analysis shows that Hmt1 loss-of-function mutants display higher steady-state tRNA abundance relative to the wild-type. Co-immunoprecipitation studies demonstrate that Hmt1 interacts with the TFIIIB component Bdp1, suggesting a mechanism for Hmt1 in modulating RNA Pol III transcription to regulate tRNA production.

**Conclusions:**

The genome-wide binding profile of Hmt1 reveals multiple potential new roles for Hmt1 in the control of eukaryotic gene expression, especially in the realm of non-coding RNAs. The data obtained here will provide an important blueprint for future mechanistic studies on the described occupancy relationship for genomic features bound by Hmt1.

## Background

Protein arginine methylation is a post-translational modification commonly found in nucleic acid-binding proteins [reviewed in [1,2]. This modification is catalyzed by a family of evolutionarily conserved enzymes called protein arginine methyltransferases (PRMTs). PRMTs can be classified into four major classes depending on the type of methylarginine generated by the enzyme. Type I, II, and III PRMTs can all catalyze monomethylation and generate monomethylarginine (MMA) on a guanidinum nitrogen of arginine residues in proteins. Type I PRMTs then catalyze the formation of asymmetric dimethylarginine (ADMA) and type II PRMTs, symmetric dimethylarginine (SDMA). Type III can only catalyze monomethylarginine while the Type IV enzyme, which has only been described in *Saccharomyces cerevisiae*[3], catalyzes monomethylation of the delta nitrogen atom of the arginine residue. Currently, eleven PRMT family members have been identified in the metazoans, with PRMT1 (Type I) and PRMT5 (Type II) being the most conserved members of the PRMT family across the eukaryotic spectrum reviewed in [4].

Hmt1 (also termed Rmt1) has been identified as the major type I PRMT in the budding yeast *S. cerevisiae* and is the functional homolog of mammalian PRMT1 [5,6]. A number of substrates have been identified for Hmt1 and knowledge of these substrates’ biological functions has helped infer Hmt1’s role as a regulator during various steps in gene expression. These substrates include histones H2A, H2B, H3, and H4 [7,8], mRNA export factors Npl3 [5] and Yra1 [9], pre-mRNA splicing factors Snp1 [10], 3’-end processing factors Hrp1 [11] and Nab2 [12], and the nucleolar proteins Nop1, Nsr1, and Gar1 [13]. Functional studies have shown that yeast mutants lacking Hmt1 display compromised intracellular protein trafficking, aberrant messenger ribonucleoprotein particle (mRNP) formation and defective silent chromatin formation [9,11,14]. The defects seen in the silent chromatin formation in Hmt1 loss-of-function mutants may be due to a change in the recruitment of other histone deacetylases to these regions, thereby altering the histone modification status at such regions [15]. Moreover, recent data from large-scale, synthetic genetic array (SGA) experiments reveal new, potential functions for Hmt1 in a cell [15-17].

Genome-wide location analysis is a powerful method that allows one to comprehensively identify direct and indirect protein-nucleic acid interactions across a specific genome *in vivo*, by combining the method of chromatin immunoprecipitation followed by hybridization to microarrays reviewed in [18,19]. For yeast, this approach has been utilized to study both DNA- and RNA-binding proteins [20-23]. For example, genome-wide location analyses of basal transcription factors have provided important information on the marking of promoter regions within a eukaryotic transcription circuit [24,25]. Additionally, the ability to use antibodies directed against specific histone modifications has enabled the creation of chromatin signature maps marking promoters that are either active or repressed, as well as other genome elements such as enhancers and transcribed regions reviewed in [26]. Thus, this approach has helped answer important questions about where in the genome a protein physically interacts and can unravel critical details of gene regulation within a cell.

In this study, we have comprehensively mapped the genome-wide occupancy for Hmt1 using NimbleGen high-resolution tiling microarrays. Based on the profile of genomic features bound by Hmt1, we found that Hmt1-binding sites are located at a number of ORFs as well as noncoding RNA (ncRNA) genes such as tRNA and snoRNA genes. To probe the potential functional consequence of Hmt1-occupancy at tRNA genes, we assayed the steady-state levels of various tRNAs in Hmt1 loss-of-function mutants and found their levels to be elevated when compared to wild-type cells. This positive change in the tRNA abundance in the Hmt1 loss-of-function mutants is likely due to a changed transcriptional output by RNA polymerase III (RNAPIII) rather than a change in the tRNA maturation process. Lastly, coimmunoprecipitation experiments show an association between Hmt1 and the TFIIIB component Bdp1, which identifies a potential molecular link by which Hmt1 is recruited to tRNA genes. Together, these results provide a global view for Hmt1 function within the cell and reveal a novel role for Hmt1 in regulating tRNA biogenesis.

## Results

### Yeast genomic features bound by Hmt1

Hmt1 has been previously shown to be cotranscriptionally recruited to genes [9] and Hmt1 loss-of-function mutants display decreased levels of dimethylated arginine 3 of histone H4 (H4R3) at silent chromatin regions [14]. Together, these observations demonstrate the significance of Hmt1’s interaction with the genome in facilitating its biological roles within a cell. While we have previously carried out genome-wide location analysis for Hmt1 in the past [9], that experiment used cDNA microarrays. Thus, there remains a lack of knowledge with respect to other genomic features that Hmt1 may interact with, such as autonomous replicating sequences (ARSs) or non-coding RNA (ncRNA) genes, which are not represented on the cDNA microarray platforms. To address this gap in knowledge for Hmt1’s association with the genome, we have comprehensively mapped the genome-wide occupancy of Hmt1 using NimbleGen’s high-resolution yeast tiling microarrays. These microarrays gave us the resolution and coverage needed for fully assess Hmt1’s genome-wide occupancy. After acquiring the data, ACME peak finding was used to analyze individual microarray data sets. We then used custom PERL scripts to integrate our biological triplicate datasets and to identify Hmt1-binding sites. Using this information, we were able to identify genomic features that are enriched for Hmt1-binding sites in our data sets and extracted peak sequences for analyses such as motif discovery.

**Figure 1:**
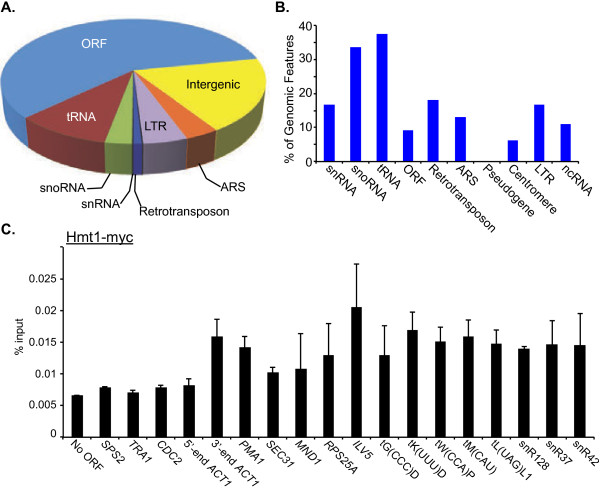
**Genome-wide binding profiles of Hmt1. A)** Pie chart showing the fraction of Hmt1 binding sites that mapped to the indicated genomic feature. **B)** The percentage of each genomic feature bound by Hmt1 as compared to the total number of that feature in the nuclear genome. **C)** Directed ChIP validation of genes bound by Hmt1. ChIP signals were normalized to “No ORF”, which is an intergenic region on the right arm of Chromosome IV [68]. *TRA1, CDC2,* and *SPS2* were negative controls. The use of *ACT1* was to demonstrate the enrichment of Hmt1 occupancy within specific regions of a gene body. Lastly, a number of tDNAs binding sites were confirmed.

Overall, a total of 1012 Hmt1 binding sites (with a peak size of 250 bp) were found to have passed the cutoff criteria for statistical significance (see Additional file 1: Table S1). Approximately 71% of these binding sites map to regions within or proximal to an annotated ORF (within 125 bp of the peak mid-point) (Figure 1A). However, this represents only 10% of all ORFs within the genome (Figure 1B). Using Gene Ontology enrichment analysis [27], we found that ORFs bound by Hmt1 are enriched for genes involved in translation (such as translational elongation, ribosomal protein genes, cytosolic ribosome, etc) and in rRNA maturation pathways (Table 1). Hmt1-binding sites also map to a number of ncRNA genes, including tRNAs, snRNAs, and snoRNAs, as well as ARSs (Figure 1A). The percentage of total features within the yeast genome that are bound by Hmt1 was calculated for each of the annotated genomic element classes (Figure 1B). This analysis revealed that Hmt1 binds to approximately 40% of all nuclear-encoded tRNA genes, as well as nearly 35% of all snoRNAs.

**Table 1:** GO term analysis for gene functional groups enriched in Hmt1-binding sites

**Adj. *p*-value**	**GO attribute**	**Description**
<0.001	6414	translational elongation/protein synthesis elongation
<0.001	30533	triplet codon-amino acid adaptor activity/tRNA/transfer RNA
<0.001	16283	eukaryotic 48S initiation complex
<0.001	16072	rRNA metabolism
<0.001	16282	eukaryotic 43S preinitiation complex/eukaryotic 43S
<0.001	9451	RNA modification
<0.001	5843	cytosolic small ribosomal subunit/40S ribosomal subunit/cytosolic small ribosomal subunit
<0.001	30563	snRNA 2′ -O- ribose methylation guide activity
<0.001	17069	snRNA binding
<0.001	30561	RNA 2′ -O- ribose methylation guide activity
<0.001	30566	snRNA modification guide activity
<0.001	154	rRNA modification
<0.001	5842	cytosolic large ribosomal subunit/60S ribosomal subunit/cytosolic large ribosomal subunit
<0.001	30555	RNA modification guide activity
<0.001	30529	ribonucleoprotein complex/RNP

To validate our high-resolution ChIP-chip data of Hmt1, we used directed ChIP (ChIP-qPCR) to verify a number of the identified binding sites. In each case, we have identified Hmt1 enrichment at the regions tested (Figure 1C). These features include specific regions within a few ORFs and ncRNA genes. As a negative control, we measured Hmt1 enrichment at regions not found in our Hmt1-bound dataset, such as *TRA1* and *SPS2* (Figure 1C). The results from our ChIP validation experiments provide additional confidence that Hmt1 binds a number of genomic features previously unknown, especially tRNA genes.

### Characteristics of Hmt1-binding across ORFs

**Figure 2:**
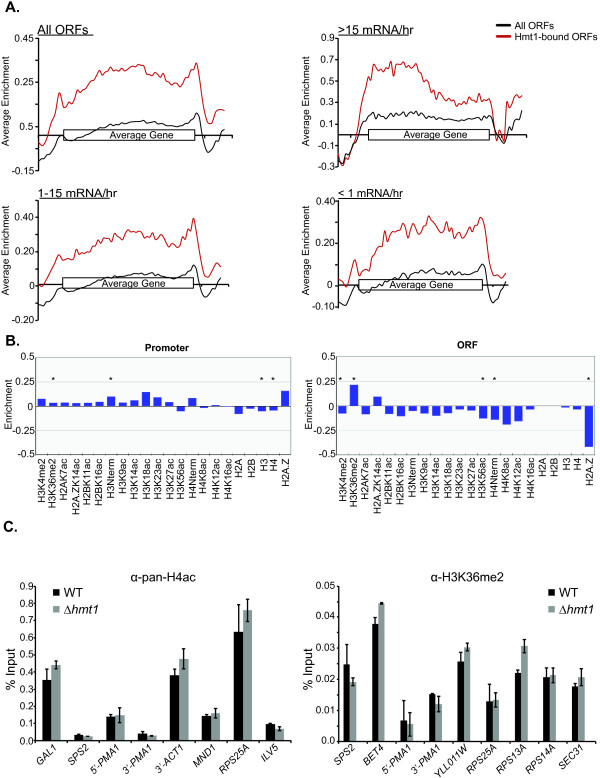
**Hmt1-binding sites are enriched at ORF genes**. **A)** Average Hmt1 enrichment profiles for all ORFs bound (“ALL ORFs), ORFs with high- (“≥ 15 mRNA/hr”), medium- (“1-15 mRNA/hr”), and low- (“ ≤ 1 mRNA/hr”) transcriptional frequencies. **B)** Histone enrichment/depletion calculations of promoters and genes for Hmt1-bound ORFs from ChromatinDB. **C)** Directed ChIP to assess the role of Hmt1 on the levels of pan-H4ac and H3K36me2. Representative ORFs bound-by Hmt1 were assayed for the level of the specified histone modification in WT and *hmt1-*null cells.

Previously, we have shown that Hmt1 is cotranscriptionally recruited to the actively transcribing gene *GAL10*, with a preference at the 5’-end to the middle of the *GAL10* ORF [9]. To examine the relationship between Hmt1 occupancy and gene expression, we assigned ORF-encoding genes to three different classes based on their transcriptional frequency [28]. We generated a baseline Hmt1 signal for all genes that fall under each of the three different classes as described previously [29] (Figure 2A, black lines in each panel), then we plotted the average Hmt1-enrichment for the set of ORFs that we classified as bound by our binding site annotations (Figure 2A, red lines in each panel). For highly transcribed genes (≥ 15 mRNAs/hr), such as those involved in translation or ribosomal protein genes, there is an obvious enrichment of Hmt1 from the 5’-end to the middle of these highly transcribed genes (Figure 2A, upper right panel, red line). This occupancy trend is similar to what we had previously observed for Hmt1 on the *GAL10* gene [9]. To our surprise, we observed a different trend for Hmt1 occupancy across ORFs with medium and low transcriptional rates (Figure 2A, lower right and lower left panels). In general, Hmt1 occupancy is evenly distributed across an ORF within these two transcriptional classes, with the exception of a slightly higher occupancy at the 3’-end of these ORFs. This is more apparent for the gene class with medium transcriptional frequency (1 to 15 mRNAs/hr) (Figure 2A, lower right panel, gray line).

Hmt1 methylates arginine 3 on histone H4 (H4R3) *in vitro*[7] and mutants lacking Hmt1 or its catalytic activities display decreased levels of dimethylated H4R3 at silent chromatin regions [14]. Since mutants lacking Hmt1 do not show a significant decrease in bulk dimethylated H4R3 [7], it is possible that Hmt1’s effects on chromatin may only occur within a specific chromosomal context, such as those in the silent chromatin regions. Nevertheless, PRMT1 (the mammalian homolog of Hmt1)-catalyzed H4R3 methylation has been demonstrated as a key, initial histone modification that is important for subsequent histone modifications [30]. As such, we wanted to determine if Hmt1-bound ORFs displayed enrichment or depletion for specific histone modifications. Using ChromatinDB, we calculated the enrichment or depletion of 17 different histone modifications and the five core histones for the Hmt1-bound ORFs (Figure 2B) [31]. ChromatinDB calculates the enrichment/depletion in gene bodies as well as promoter regions across the yeast genome [31]. Analysis of the Hmt1-bound genes revealed that promoter regions were enriched for H3K36me2 and pan-H3 acetylation, while being depleted for H3/H4 occupancy (Figure 2B, promoter panel). H3K4me2, pan-H3 acetylation, H3K56ac, and H2A.Z occupancy are depleted, and H3K36me2 is enriched across gene bodies (Figure 2B, ORF panel).

To assess whether Hmt1 regulates the modification of histones based on our ChromatinDB analysis, we performed directed ChIP using antibodies against pan-acetyl H4 and H3K36me2 in wild-type and *hmt1-*null cells to measure the levels of these modifications in ORF-gene bodies since we find the majority of Hmt1 enrichment here (Figure 2A). We tested the ORFs that were bound by Hmt1 and representative of the histone modification trend (i.e. enriched/depleted for a specific modification) based on the database analysis. With respect to pan-acetyl H4, we do not observe any changes in the level between wild-type and *hmt1-*null cells (Figure 2C, α-pan-H4ac panel). When we compared the directed ChIP results for H3K36me2 between wild-type and *hmt1-*null cells, we found the majority of the ORFs we tested do not change, but there was a slight increase in two loci – *BET4* and *RPS13A* (Figure 2C, α-H3K36me2 panel). These results suggest that the absence (in the case of pan-H4ac) or presence (in the case of H3K36me2) of these histone modifications may regulate the recruitment of Hmt1 as opposed to Hmt1 regulating their levels in ORF gene bodies.

### Analysis of Hmt1-bound sequences for motif discovery

Because we were able to obtain detailed sequence-specific information from the probes used in our high-resolution microarray, we applied a number of motif finding algorithms (AlignACE [32], MEME [33], WEEDER [34], and MDScan [35]) to determine whether there exists a motif within the Hmt1 binding sites. While our analyses revealed enriched motifs, none of these appear to be of obvious biological significance (data not shown). This was not surprising to us, as Hmt1 has no reported DNA binding activity and is likely to associate within the proximity of the DNA via its interaction with DNA-binding proteins. This type of indirect interaction likely will impede finding true motif signals. However, we also took a reciprocal approach in which we searched all of the Hmt1-binding sites for known transcription factor sequence motifs using previously generated data [25,36,37]. Using the 250 bp window for Hmt1-binding sites in our search and a negative control of 10,000 randomized yeast genome sequences of the same size, we carried out an analysis for the enrichment of known TF binding sites within our Hmt1-binding sites. Our analyses did reveal a number of over-represented TF-binding sites within the Hmt1-binding sites that passed statistical significance cutoff (Additional files 2: Table S2). A few of the most enriched TF-binding sites were for transcription factors Cat8, Tbp1, Dal81, Ume6, and Mig1. Except for Tbp1, which is the general, TATA-binding protein that interacts with other factors to form the preinitiation complex at promoters [38], the other four transcription factors are all involved in specific metabolic pathways [39-41] or life cycle stages (such as Ume6) [42]. Thus, it is possible that Hmt1 functions together with these transcription factors to help regulate the expression of genes involved in these pathways.

### Hmt1-binding is enriched at regions proximal to tRNA genes

**Figure 3:**
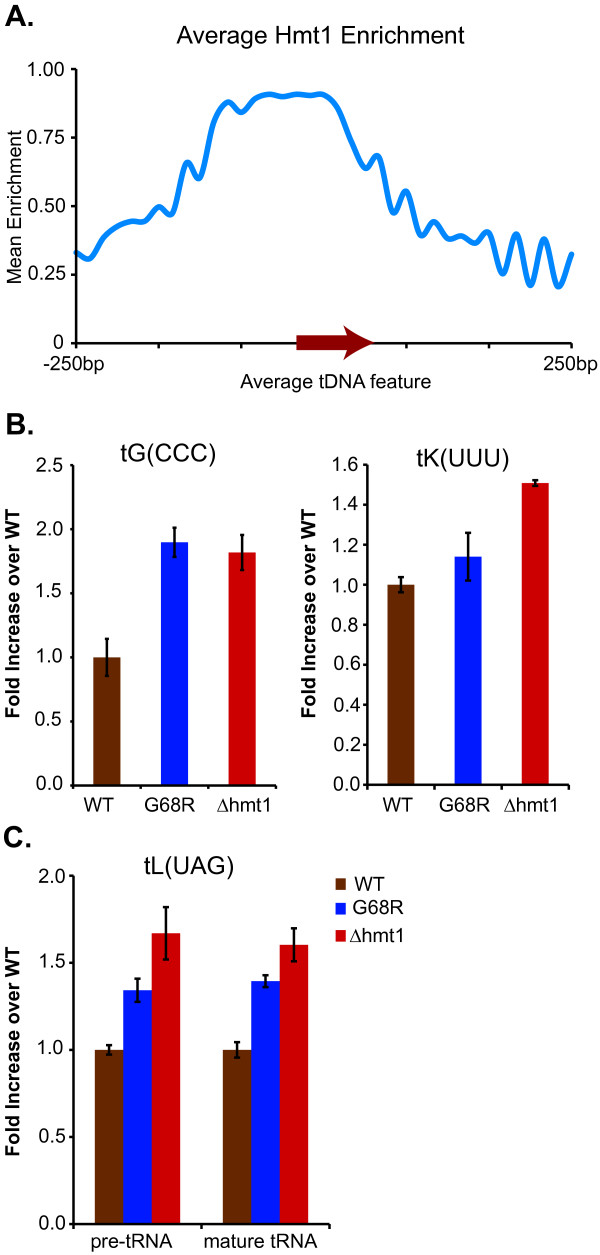
**Hmt1-binding sites are enriched at tRNA genes**. **A**) The average Hmt1-enrichment pattern across all Hmt1-bound tDNA features. **B**) Hmt1 loss-of-function mutants displayed increased tRNA abundance. Bar graph showing RNA hybridization analysis results on the relative abundance of mature tRNA tG(CCC) and tK(UUU) in the Hmt1-null (Δhmt1) and Hmt1-catalytically inactive mutants (G68R). Signals were normalized to the 5.8S rRNA (RNAPI transcript). **C**) Both pre- and mature tRNA abundance is increased in Hmt1 loss-of-function mutants. Bar graph showing RNA hybridization analysis results on the relative abundance of pre- and mature tRNA tL(UAG) in the Hmt1-null (Δhmt1) and Hmt1-catalytically inactive mutants (G68R). Signals were normalized to the 5.8S rRNA (RNAPI transcript).

From our ChIP-chip data, we found that Hmt1-binding is enriched across the 5’-regions that are proximal or within the gene body of the nuclear-encoded tRNA genes (Figure 3A). Unlike the data from our Hmt1-bound ORF analyses for genes with medium- and low-transcriptional rates (Figure 2), we do not find any special enrichment for Hmt1 near the 3’-end of the gene body (Figure 3A). Given the preferred Hmt1 occupancy at an average tDNA gene, it implicates a potential role for this enzyme in modulating tRNA biogenesis, potentially either at the level of transcription or via processing, as transcribed tRNAs undergo many processing events such as base modification and splicing during their maturation.

Interestingly, we found that the majority of Hmt1 occupancy at tDNAs did not follow an all or none trend when we examined the binding of Hmt1 to the entire set of alleles for a specific tRNA gene species. For example, we found Hmt1 occupancy at five of seven tDNAs encoding tK(UUU). However, in some instances such as tDNAs that encode tG(CCC), we did find Hmt1 occupancy at both of the tDNA alleles. As a way to determine whether Hmt1-binding may be correlated to the codon usage, we calculated the correlation coefficient for Hmt1-binding and specific codon usage. However, such analysis did not reveal any credible candidates (data not shown), suggesting that Hmt1-binding to these tDNA genes is likely a general phenomenon.

### Mutants lacking Hmt1 or its catalytic activity display increased tRNA abundance

To determine if the observed enrichment of Hmt1-binding around tRNA genes had any functional significance, we used RNA hybridization assays to compare the levels of steady-state tRNA abundances between wild-type and Hmt1 loss-of-function mutants. We checked the matured form of tRNA tG(CCC), which is encoded by two tDNA alleles, both of which were bound by Hmt1 based on our analysis. In both Hmt1 loss-of-function mutants, there is an approximately two-fold increase in the levels of mature tG(CCC) tRNA when compared to that of the wild-type (Figure 3B, tG(CCC) panel). We next checked another tRNA gene, tK(UUU), that had five out of seven tDNA alleles bound by Hmt1. In this case, an increase in tRNA abundance is still observed, but the magnitude of the increase is not as significant as that of tG(CCC) (Figure 3B).

During tRNA biogenesis, various processing steps must take place in order to generate final, mature tRNAs. Hmt1 has been previously implicated in mRNP dynamics and many of its substrates are mRNA processing factors. Thus, it is possible that the change in the tRNA abundance observed in the Hmt1 loss-of-function mutants may be due to a role for Hmt1 in the tRNA processing steps. To test this possibility, we used a previously published probe that would hybridize equally to both the pre-processed form and the mature form of tL(UAG), which had one out of three tDNA alleles bound by Hmt1. In this case, we observe an increase in the pre-tRNA in the Hmt1 loss-of-function mutants, similar to that of the mature tRNA changes (Figure 3C). Moreover, the ratio of pre-tRNA to total and the ratio of mature tRNA to total were not changed in these mutants. This comparison suggests that the increased tRNA abundance observed for the Hmt1 loss-of-function mutants is likely due to a change in the overall transcriptional output of tRNA transcripts observed in these mutants.

### Hmt1 physically associates with TFIIIB component Bdp1

**Figure 4:**
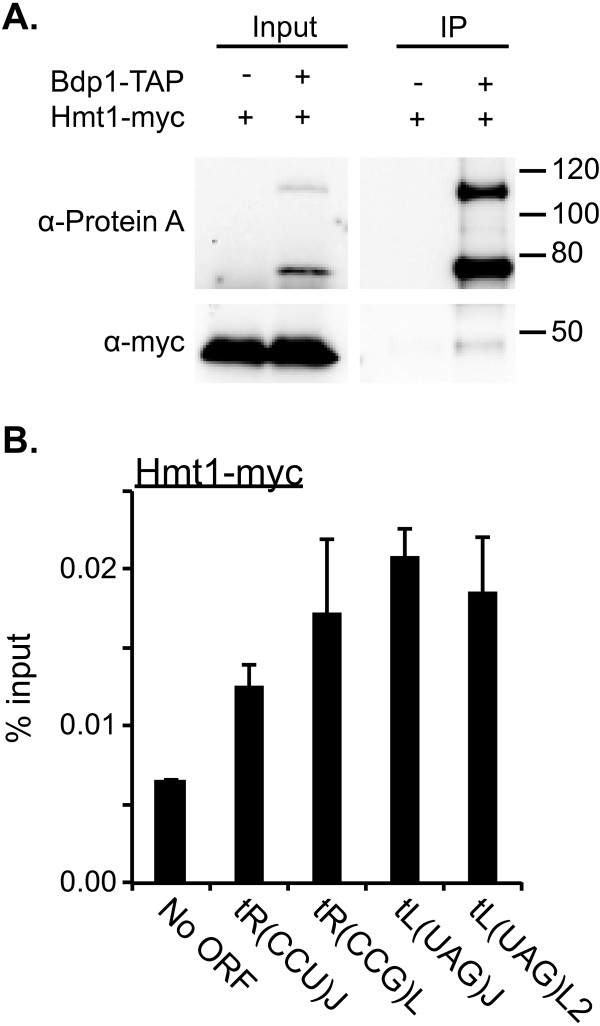
**Hmt1 physically associates with TFIIIB component Bdp1. A)** Affinity purification of Bdp1-TAP results in the co-purification of Hmt1-myc, as shown by immunoblotting with α-myc (for Hmt1) and α-Protein A (for Bdp1). **B)** Directed ChIP shows Hmt1 binding on tDNAs tR(CCU)J, tR(CCG)L, tL(UAG)J, tL(UAG)L2. ChIP signals were normalized to “No ORF”, which is an intergenic region on the right arm of Chromosome IV [68].

Knowing that Hmt1 is bound at tRNA genes and that Hmt1 loss-of-function mutants display an increased tRNA abundance, there exists a potential regulatory role for Hmt1 in repressing RNAPIII transcription at tRNA genes. Given that the average enrichment of Hmt1 is mostly at the 5’-end and throughout the gene body, it is possible that Hmt1 may exert its control on tRNA biogenesis through some action on the components of the RNAPIII machinery. Interestingly, a recent proteomic profiling study for Hmt1 in our laboratory had identified the TFIIIB component Bdp1 as a potential physical interactor of Hmt1 (data not shown). Bdp1 is one of the three components of TFIIIB [43] and has been implicated to function as a scaffold for TFIIIB-DNA assembly [44]. To verify this physical association between TAP-tagged Hmt1 and Bdp1, we performed a co-immunoprecipitation with a reversed bait and target. In this case, we purified TAP-tagged Bdp1 and the purified eluates were assayed for the presence of myc-tagged Hmt1 by immunoblotting. Our immunoblotting data (Figure 4A) show that purification of TAP-tagged Bdp1 results in co-purification of Hmt1. As a negative control, we have carried out a parallel purification using lysates from a yeast strain that lack TAP-tagged proteins. Thus, we have confirmed the physical association between TFIIIB component Bdp1 and that of Hmt1.

It is likely that the Hmt1 recruitment to tRNA genes occurs via its physical association with Bdp1. While our ChIP-chip data only show Hmt1-binding to approximately 40% of all tDNAs, the observation of Hmt1’s physical association with Bdp1 suggests that Hmt1 is binding to the majority of the tDNAs, if not all of them. This is based on the fact that TFIIIB occupancy is found across all tDNAs from a number of genome-wide location analyses in *S. cerevisiae*[45-47]. Therefore, it is likely that our stringent criteria for classifying what is considered an Hmt1-bound locus excludes some true Hmt1-bound sites at these tDNAs. To test this hypothesis, we used ChIP to determine the association of Hmt1 at four different tDNAs, which were not bound by Hmt1 based on our ChIP-chip data. As shown in Figure 4B, however, there is an enrichment of Hmt1 at all four tDNAs examined, suggesting that indeed there may be some Hmt1-binding sites that are excluded in our final ChIP-chip data set because of high stringency. This also means that we are highly confident of the Hmt1-binding sites determined from our original analysis.

## Discussion

In this study, we applied genome-wide location analysis to uncover genomic features previously not known to associate with Hmt1, the yeast homolog of human PRMT1. To this end, we identified binding of Hmt1 to a number of ncRNA genes such as tRNA and snoRNA genes. Enrichment of Hmt1 binding at these genomic features directly implicates a regulatory role for this protein in biological processes or pathways involving these genomic features. We validated the biological significance of Hmt1 binding at the tRNA genes using RNA hybridization analyses in assaying Hmt1 loss-of-function mutants, which display an increase in the steady-state levels of pre- and mature tRNA when compared to the wild-type cells. This increase is likely attributed to a change in the overall transcriptional output by RNAPIII, as opposed to a defect in the maturation process of tRNAs. The physical association between Hmt1 and TFIIIB component Bdp1 provides a likely explanation for Hmt1’s association at many of the tRNA genes in our ChIP-chip data.

Hmt1 has previously been demonstrated to play a role in the recruitment of mRNP components such as pre-mRNA splicing factors [48] and mRNA export factors [9]. Furthermore, it regulates transcriptional elongation and termination via methylation of hnRNP and mRNA export factor Npl3 [49]. Thus, it was not surprising for us to find the majority of Hmt1 binding sites fall proximal to or within an ORF. GO-enrichment analysis indicates Hmt1 binding to genes involved in translation, suggesting a potential role for this enzyme in modulating the expression of these genes. Transcriptional profiling experiments carried out previously on both null and catalytically-inactive mutants of Hmt1, however, show no significant changes in the abundance of these transcripts from the wild-type cells [9]. Based on these observations, one possible explanation is that Hmt1’s effects on these genes can only be seen under a specific circumstance, such as a change in the environmental condition or an exposure to certain stress agent. This scenario would support the observed growth phenotype for the Hmt1-null mutants as they display no distinguishable changes in their growth rate from wild-type cells when grown in a rich media, such as YPD [6]. Transcription of *HMT1* is repressed in response to heat shock when compared to steady-state growth rate conditions [50], further suggesting that Hmt1 is needed the most only under a certain growth condition. It is interesting to note that Hmt1 has different occupancy patterns across genes with different transcriptional rates, as this observation demonstrates a potential for different modes of regulation during gene expression by Hmt1. There are two functional implications of Hmt1’s enrichment across the various transcriptional frequency gene classes – first, the highly transcribed gene class contains many of the ribosomal protein genes that encompass many intron-containing genes and Hmt1 has been shown to be more enriched at the 5’-end of these genes. Since Hmt1 affects the co-transcriptional recruitment of pre-mRNA splicing factors [10], Hmt1’s enrichment across this region may reflect its role in regulating this process. Second, Hmt1’s enrichment across all ORFs shows a “spike” at the 3’-end of the genes, with this trend more pronounced in gene classes with medium and low transcriptional frequencies. This enrichment trend may reflect the association of Hmt1 with known substrates involved in 3’-end processing of a transcript, such as Yra1, Hrp1, and Nab2. Thus, these observations suggest that Hmt1 may methylate its substrate during transcription rather than prior to.

Hmt1 methylates H4R3 *in vitro*[7] and loss-of-function mutants of Hmt1 display decreased dimethylated H4R3 levels across silent chromatin regions [14], despite the observation that bulk dimethylated H4R3 is unchanged in *hmt1* mutants [7]. Thus, Hmt1-catalyzed H4R3me2 in *S. cerevisiae* is associated with gene silencing. H4R3 methylation by PRMT1 *in vivo* is required for many subsequent histone modifications in mammalian cells [30]. It can be inferred then that Hmt1 may also play a role in modulating the levels of other histone modifications. By determining the enrichment or depletion of core histones and other histone modifications for Hmt1-bound ORFs, the data reveal that Hmt1 may coordinate regulation at the promoter with H3-specific acetyltransferases such as SAGA or NuaA3 and the Rpd3(S) complex, which is an H3K36 methyl reader [51-53]. Furthermore, our ORF-body analysis suggests that Hmt1 may coordinate the action of COMPASS (responsible for H3K4 methylation), Set2 (responsible for H3K36 methylation) as well as KDACs [54-57]. Results from our directed ChIP experiments, however, suggests that these histone modification marks may regulate the recruitment of Hmt1 to ORF-containing gene bodies instead, at least for pan-H4 acetylation and the majority of H4K36me2 tested. It is possible that the loss of Hmt1 alone is not sufficient to alter these histone modification states we have identified. Rather, it may be the combination of Hmt1-deletion with the loss of another regulator that effects a significant change in histone modification levels as compared to any single loss-of-function mutant.

Perhaps the most interesting hypothesis we obtained from analyzing our ChIP-chip data is a role for Hmt1 in the biogenesis of tRNAs. In eukaryotic cells, tRNAs are classified on the basis of their anticodon, of which there are 42 different tRNA species in the *S. cerevisiae*[58,59]. Furthermore, the actual number of different tRNA species is greater in some cases because there can be species with identical anticodons but sequence differences in the tRNA body [60]. In *S. cerevisiae*, each tRNA species can be encoded by several genomic copies (tDNAs), of which there are a total of 274 nuclear-encoded tDNAs within the genome [59]. Hmt1 binding is enriched at many of these tDNA alleles and mutants lacking Hmt1 or its catalytic activity display higher levels of the corresponding tRNA abundance. This overall change is likely attributed to a defect in the output rather than the processing during tRNA biogenesis, as both pre- and mature tRNAs increase in a similar fashion in the Hmt1 loss-of-function mutants. If the change in the mature tRNA abundance was attributed to a defect in tRNA processing in the Hmt1 mutants, we would expect the ratio (pre- to mature tRNA) of signals obtained from our RNA hybridization analysis to reflect such.

Transcription of tRNAs is accomplished by RNAPIII and this process requires two gene-internal promoters, boxA and boxB reviewed in [61,62]. During tRNA gene transcription, these internal promoters are bound by the hetero-heptameric complex TFIIIC. TFIIIC recruits the hetero-trimeric TFIIIB (which consists of Bdp1, Brf1, and TBP) to the upstream region of the tDNA. TFIIIB, in turn, recruits RNAPIII to prompt transcriptional initiation. The physical interaction between Hmt1 and Bdp1 provides a probable mechanism for how Hmt1 may be recruited to these tDNAs. Additionally, this physical interaction gave insights into how Hmt1 may affect the overall tRNA abundance through its ability to regulate RNAPIII transcription of tRNA genes. While the precise molecular mechanisms by which Hmt1 accomplishes this remains to be elucidated, our data provide a valuable clue in that Hmt1 does so via its catalytic activity, based on our data from the Hmt1 catalytically inactive mutant. This points to a yet-to-be discovered substrate of Hmt1 in which its methylation may affect the transcription of tRNA genes by RNAPIII. The biological significance of this transcriptional repression by Hmt1 could reflect a way for coordinating gene expression between RNAPII and RNAPIII, given the known role of Hmt1 in coordinating mRNP biogenesis catalyzed by RNAPII. As it is advantageous for cells to efficiently utilize its resources during growth (especially in sub-optimal conditions), proper coordination would be important for not wasting any valuable resources in executing gene expression programs. Thus, it can be inferred that Hmt1’s function in balancing these gene expression programs may not be as critical when cells are growing under optimal conditions with plenty of resources, as supported by the observation that *hmt1-*null cells do not display any obvious growth defect in rich media. Further experiments are required to define the specific molecular mechanisms used by Hmt1 in this process as well as other roles Hmt1 may play in the described occupancy relationship for genomic features found within Hmt1 binding sites. In sum, our genome-wide location analysis data do reveal multiple potentially new roles for Hmt1 in the control of eukaryotic gene expression *in vivo*, especially in the realm of ncRNA biogenesis.

## Conclusions

Our data on the comprehensive mapping of Hmt1 binding sites across the yeast genome uncover many genomic features previously not known to associate with Hmt1, thereby implicating novel role of this enzyme in modulating biological processes facilitated by these genomic features. One such example we validated in this study is the tRNA gene, for which Hmt1 occupancy is enriched. The functional implication of Hmt1 binding on tRNA biogenesis is supported by the observation that binding of Hmt1 at these tRNA genes correlates with their abundance levels within a cell. At the molecular level, our data suggest that Hmt1 is recruited by Bdp1, a component of RNAPIII transcriptional machinery, to the tRNA genes. Together, these results suggest a potential role for Hmt1 in modulating tRNA biogenesis via RNAPIII transcription. Overall, our study demonstrates that knowledge of genome-wide locations for a multi-functional protein such as Hmt1 has the potential to lay the foundation for studies that reveal the factors responsible for recruiting Hmt1 to a specific genomic feature. Given the level of conservation between Hmt1 and human PRMT1, it is likely that some of our observations have implications for studies in higher eukaryotes.

## Methods

### Yeast strains used in this study

All yeast *Saccharomyces cerevisiae* strains used in this study are listed in Table 2 of the section. All strains were grown at 30°C on YEPD medium (1% yeast extract, 2% bactopeptone, 2% D-glucose, w/v) unless otherwise stated

**Table 2:** Yeast strains used

**Strain #**	**Genotype**	**Ref**
MYY65	*ade2-1 trp1-1 can1-100 leu2-3,112 his3-11,15 ura3 GAL+ psi+ HMT1::9MYC::TRP*	[9]
BY4741	*his3∆ 1 leu2∆ 0 met15∆ 0 ura3∆ 0*	[70]
MYY649	*his3∆ 1 leu2∆ 0 met15∆ 0 ura3∆ 0 Δhmt1::KANMX6 hmt1-G68R::LEU2*	This Work
MYY432	*his3∆ 1 leu2∆ 0 met15∆ 0 ura3∆ 0 ∆hmt1::KANMX6*	This Work
MYY1200	*his3∆ 1 leu2∆ 0 met15∆ 0 ura3∆ 0 BDP1::TAP::HIS3 HMT1::9MYC::NAT*^*R*^	This Work
MYY1097	*his3∆ 1 leu2∆ 0 met15∆ 0 ura3∆ 0 HMT1::9myc::NAT*^*R*^	This Work

### Hmt1 ChIP-chip experiment

The ChIP-chip procedure was performed as described previously [9]. Briefly, formaldehyde was used to crosslink myc-tagged Hmt1 and associated nucleic acids in vegetatively growing yeast cells. Upon harvesting of the crosslinked yeast cells, soluble chromatin was prepared by sonication to generate chromatin fragments on an average of 300bp. Following sonication, the chromatin was subjected to immunoprecipitation using 9E11 anti-myc antibody (ThermoFisher) to enrich for Hmt1-associated DNA. After reversal of the crosslinks, the enriched DNA was amplified linearly by ligation-mediated PCR. As a control, a sample was prepared from total DNA (without undergoing immunoprecipitation). The resulting, enriched DNA samples were sent to NimbleGen for hybridization to high-resolution *S. cerevisiae* tiling arrays (385K arrays), followed by the scanning and pre-processing of the array data. All the microarray data were deposited in NCBI's GEO database, Accession: GSE40505

For data analysis, the “Algorithm for Capturing Microarray Enrichment” (ACME) [63] was used to determine the sequence regions that were bound by epitope-tagged Hmt1 on the chromosome. ACME, which does not assume that log_2_ ratio data are Gaussian-distributed like other algorithms, makes only two assumptions: (1) that data are enriched for signal in the positive direction (“one-tailed”) and (2) that the real signal will be represented by multiple probes that are genomically located close to one another (“neighbor effect”). Despite its simplicity, ACME has been proven to be quite robust at detecting true signal from noise [64]. For our analysis, the raw array data was normalized using bi-weight mean by NimbleScan Version 2.1 software (NimbleGen Systems).

To identify potential sites of enrichment, multiple sliding windows of 600–, 800– and 1,000–bp were separately employed to move stepwise along the tiled region, centering at every probe. Hybridization signals of probes within each window were tested by χ^2^ analysis to determine if the window contains a higher than expected number of probe signals above the defined threshold. The resulting *p*-value that is associated with each averaged data point was used for the selection of significant regions. To minimize false positives, we also employed a two-state hidden Markov model in an independent parallel analysis of the data set [65], in which two distributions were constructed to characterize binding and non-binding probe intensities, to determine regions of significance. The common significant regions from the above two methods were considered to be true signals. The three biological replicate sets of Hmt1 binding site data generated were then coalesced by assessing the binding-site midpoint distances and merging peaks from at least two datasets that were less than 125 bp apart. Peaks were then either extended or truncated to 250 bp (midpoint +/- 125 bp) for uniformity.

### Bioinformatics analysis of Hmt1 binding sites

Annotations were done using PERL scripts and a dump of the SGD genome database feature file (downloaded on 09/2010). Average feature plots were generated as previously described [29]. Briefly, ORF sets were partitioned into 50 bins and the probes were summed and averaged for each bin. To minimize sampling errors, the average bin size for a class of ORFs was used to create bins in the upstream and downstream regions, which were fixed at 250 bp. For motif searches, Hmt1-binding sequences were first masked (A.F.A. Smit, R. Hubley & P. Green, unpublished data. Current Version: open-3.3.0) and then analyzed using MEME [33], AlignACE [32], Weeder [34], and MDScan [35] with their default settings.

Histone modification enrichment/depletion analysis was carried out at the ChromatinDB website (http://www.bioinformatics2.wsu.edu/cgibin/ChromatinDB/cgi/visualize_select.pl)[31]. The input list of Hmt1-bound ORFs was used for nucleosome occupancy-normalized histone modifications, with a *p*-value cutoff of 0.001, and Bonferroni multiple hypothesis test correction [31].

To search whether the identified Hmt1 binding sites are enriched for other transcription factor (TF) binding sites, we preformed the following analysis: A size of 250 bp is assigned for each peak and used to determine which TF binds the peak. The CLOVER program was used with 184 fungi position weight matrixes (PWMs) from public domain information on TFs [66] to predict TF binding sites. Parameters of CLOVER [67] were set for 1000 randomizations and a *p*-value threshold of 0.05 using background sequences of 10,000 random DNA fragments of the same size generated from yeast chromosomes 1, 3, 5, 7, and 9 (2000 fragments from each chromosome).

### Chromatin immunoprecipitation (ChIP)

ChIP procedures were performed as described previously [68] with at least duplicate biological samples (Hmt1-myc; n=2, pan-acetyl-H4 and H3K36me2; n=3). For each biological replicate, qPCR was performed in three technical replicates. For qPCR, 2 μl of DNA sample (input or IP) was used in 20 μl reactions with 250-500nM final concentration of each primer and Bio-Rad Sso Advanced SYBR Green Supermix or Invitrogen Power SYBR Green PCR Master Mix. Table 3 shows the primers used in all qPCR experiments in this study. For each immunoprecipitation, 10 μl of monoclonal α-myc (9E11, Thermo-Fisher), or 5 μl of α-H3K36me2 (EMD Millipore cat#07-369), or 5 μl of α-pan-acetyl-H4 (Upstate Biotechnology, cat#06-598) was pre-coupled to 40 μl of Protein-A sepharose beads.

**Table 3:** Oligonucleotides used in this study

**NAME**	**Sequence (5′ –> 3′)**	**Reference**
5′ –PMA1(sense)	CGACGACGAAGACAGT GATAACG	This study
5′ –PMA1(anti-sense)	ATTGAATTGGACCGACGAAAAACAT AAC	This study
3′ –PMA1 (sense)	AAA CAA CCA GCT TCG GTG TGT GTG	This study
3′ –PMA1 (anti-sense)	TAG GAG CCA ACA AGA ATA AGC CGC	This study
ILV5 (sense)	ATT GGG TAC CAG TCC AAA GCA CCT	This study
ILV5 (anti-sense)	ACC GTC GAA GAA GCT ACC CAA TCT	This study
RPS25A (sense)	TGG GTA GCT AGA TCT GTG CTA TGG	This study
RPS25A (anti-sense)	GAA ATA GTG ACT GGC CAA TCC AGG	This study
GAL1 (sense)	TGCTAGATCGCCTGGTAGAG	[72]
GAL1 (anti-sense)	GCAAACCTTTCCGGTGCAAG	[72]
MND1 (sense)	CCATCATGGTACACAGGACTGAACA	This study
MND1 (anti-sense)	GGAGTGATACTGTCTGTCTCTTTGGC	This study
BET4 (sense)	CGACAGCAAGTTCAGTTTGCCACA	This study
BET4 (anti-sense)	AGCGAGTTTGGCTTCTGAGTTGGA	This study
SEC31 (sense)	CCAAATGATATCGCCCAGAGGAATG	This study
SEC31 (anti-sense)	CCGGCAGCCAAGTACAAAGTCAAA	This study
RPS13A (sense)	CAGAAAGCACTGGCAAGAACGTGT	This study
RPS13A (anti-sense)	TCTGCCAGCTCTGACCTTTCTGTT	This study
RPS14A (sense)	TGGTAAGGAAACCATCGCCAGAGT	This study
RPS14A (anti-sense)	TAACGTGAACGGCAGTGATACCGA	This study
YLL011W (sense)	AGCCAGTTTCCGATCTATCATGGG	This study
YLL011W (anti-sense)	GGCCTCCATTGGATTCCAGCAAAT	This study
snR37 (sense)	TGGAGTGTGAGTGATGAGGAGCTT	This study
snR37 (anti-sense)	GGAGTAGTCAAAGTTCATTCAGCTATGGG	This study
snR42 (sense)	CTGTTGGTGCTGAGGTAATCCATC	This study
snR42 (anti-sense)	ACCTCAGGTCATCACCATTTCATGGG	This study
ACT1 5′ end (sense)	GTCCCAATTGCTCGAGAGATTTCTC	This study
ACT1 5′ end(anti-sense)	CATGATACCTTGGTGTCTTGGTC	This study
ACT1 3′ end(sense)	TCGAACAAGAAATGCAAACCG	This study
ACT1 3′ end(anti-sense)	GGCAGATTCCAAACCCAAAAC	This study
CDC2 (sense)	CCTGCCTTTAAGGCTTATGGA	This study
CDC2 (anti-sense)	CCACGAATAGGCTCAATAACA	This study
TRA1 (sense)	CCAATTTTTGATAAGCCACCCTGA	This study
TRA1 (anti-sense)	CGTAATTTCTAAGGTCTTGTTCTCCCA	This study
SPS2 (sense)	ACTGTCCCGTCATTGATGCGTCTC	[73]
SPS2 (anti-sense)	GGGATCGTTGCATTAGTGTTAACC	[73]
No ORF (sense)	GAAAAAGTGGGATTCTGCCTGTGG	[68]
No ORF (anti-sense)	GTTTGCCACAGCGACAGAAGTATAACC	[68]
tG (CCC) D (sense)	AAATGCGGAAGCCGGGAATCGAA	This study
tG (CCC) D (anti-sense)	GGTATTTCTTTGCGCGGTTACGGT	This study
tL (UAG) L1 (sense)	GGT TAAACCCAC CTAAATCTGACGCC	This study
tL (UAG) L1 (anti-sense)	TGGCTAACCCAATGGCTTGT	This study
tW (CCA) P (sense)	AAGCTGAGTGTCCGCTGTGATGAT	This study
tW (CCA) P (anti-sense)	TTGCAATCGAAGGGTTGCAGGTTC	This study
tK (UUU )D (sense)	TGTTGGAACGGTAAAGACCAGTGC	This study
tK (UUU) D (anti-sense)	AAAGCCGAACGCTCTACCAACTGA	This study
tG (CCC) probe	ATGCTTGGGAAGCATAAATTCTA	This study
tL (UAG) probe	AAGATATCAGAGCCTAAATCTGACG	[71]
pre-t L(UAG) probe	GATATCAGAGATTTTAGAGGTTAAATCCACCT	[71]
5.8S probe	GCGTTGTTCATCGATGC	This study
tK (UUU) probe	TGACATTTCGGTTAAAAGCCGAACGCTCTACCAAC	[71]
tR (CCG) L (sense)	AAGTACGACATCAAAGTCGCCGAG	This study
tR (CCG) L (anti-sense)	TGCTAACCATTGCACTAGAGGAGC	This study
snR128 (sense)	ACAGTATACGATCACTCAGACATCC	This study
snR128 (anti-sense)	CACGGTGATGAAAGACTGGTTCCT	This study
tR (CCU) L (sense)	CCACATTCTCTACAATATTGATTTCCATCG	This study
tR (CCU) L (anti-sense)	CCATTACGCCAACGGAACCAACTT	This study
tL (UAG) L2 (sense)	ATCGACAGCTTCACGTGCCATTTG	This study
tL (UAG) L2 (anti-sense)	CTGATATCTTCGGATGCAAGGGTTCG	This study
tL (UAG) J (sense)	GGTTAAACCCACCTAAATCTGACGCC	This study
tL (UAG) J (anti-sense)	TGGCTAACCCAATGGCTTGT	This study

### RNA hybridization analysis

Total RNA was prepared from yeast strains (Table 2) grown to early log phase (OD_600_=0.2 to 0.3), using the hot phenol method as previously described [69]. For RNA hybridization analysis, 20 μg of total RNA was resolved on an 8% PAGE-urea (7.5M) gels and then transferred to HybondN+ Nylon membranes (GE Healthcare) usingBio-Rad semi-dry transfer (150 mA for 1hr). The membranes were then UV-crosslinked with a Stratalinker using the Optimal Crosslink setting. Membranes were hybridized in 3X SSC, 1X Denhardt’s, 0.5% SDS, and 100 μg/mL sheared salmon sperm DNA overnight at 37°C with ^32^P-end labeled oligonucleotide probes (see Table 3 for sequences). After hybridization, membranes were rinsed once and then washed twice for 20 mins with 50 ml of 2X SSC/0.1% SDS buffer at 37°C. The membranes were exposed to PhosphorStorage screens, scanned with a PhosphorImager (Molecular Dynamics) and quantified using the ImageQuant software.

### Coimmunoprecipitation study between Hmt1 and Bdp1

Preparation of yeast cell lysates for co-immunoprecipitations between Hmt1-myc and Bdp1-TAP was carried out as described previously [48]. Bdp1-TAP was purified by incubating prepared yeast cell lysates with 40 μL IgG sepharose beads (GE-Amersham) for 2 hours at 4°C, followed by three washes with IPP150 buffer. Bound protein was eluted in 300 μL Elution Buffer (0.5 M Acetic Acid pH 3.5), precipitated with trichloroacetic acid (TCA) overnight at -20°C, recovered, and resolved on a NuPAGE 4-12% Bis-Tris gradient gel (Life Technologies). The immunoblot was probed with an anti-myc antibody (Santa Cruz A-14), stripped, and re-probed with an of the immunoblot α-Protein A antibody (Sigma, cat#P3775).

## Abbreviations

ACME: Algorithm for Capturing Microarray Enrichment; ADMA: Asymmetric dimethylarginine; ARS: Autonomously replicating sequence; MMA: Monomethylarginine; PRMT: Protein arginine methyltransferase; RNAPI: RNA polymerase I; RNAPII: RNA polymerase II; RNAPIII: RNA polymerase III; SDMA: Symmetric dimethylarginine; TF: Transcription factor.

## Competing interests

The authors declare that they have no competing interests.

## Authors’ contributions

EJM and MCY conceived and designed the experiments. MCY carried out the ChIP-chip experiments. EJM and ZH performed and contributed to the microarray data processing and analysis. EJM carried out additional experiments and data analysis. EJM and MCY wrote the paper. All authors read and approved the final manuscript.

## Supplementary Material

Additional file 1 Table S1 Hmt1 binding site coordinates trimmed to 250 bp. The file is in gff format, the columns are listed below: Column 1: Chromosome. Column 2: Source. Column 3: feature. Column 4: Start Coordinate. Column 5: Stop Coordinate. Column 6: Score. Column 7: Strand. Column 8: Reading Frame. Column 9: Attributes (GFF 86 kb) Click here for file

Additional file 2 Table S2 TF motif search. Results from transcription factor motif search. Transcription factors are listed on left, followed by number of Hmt1 peaks found to contain the TF’s motif. Followed by three different methods of background motif estimation. 1) Sequence shuffle of Hmt1 peaks, 2) *S. cerevisiae* promoter sequences (-500bp to +100bp of every ORF) and 3) 3’-UTR sequences for each ORF. (XLSX 53 kb) Click here for file
